# Fuzzy random evaluation of creep model of frozen soft soil in metro tunnel construction using artificial ground freezing technique

**DOI:** 10.1038/s41598-023-36322-x

**Published:** 2023-06-10

**Authors:** Yafeng Yao, Yan Zhu, Dejian Shen, Zhemei Zhang, Wei Wang

**Affiliations:** 1 School of Civil Engineering, Nantong Vocational University, Nantong, 226001 China; 2 School of Civil and Traffic Engineering, Hohai University, Nanjing, 210098 China; 3 Anhui Key Laboratory of Building Structure and Underground Engineering, Anhui Jianzhu University, Hefei, 210037 China; 4 College of Civil Engineering, Tongzhou Secondary Vocational School, Nantong, 226399 China

**Keywords:** Civil engineering, Mechanical engineering, Applied mathematics, Computational science, Computer science

## Abstract

Mastering the creep characteristics of artificial frozen soil and scientifically evaluating the creep model is an important guarantee for the safety of subway tunnel freezing construction. Base on the construction of Nantong metro tunnel, the uniaxial compressive strength tests of the artificially frozen soft soil were carried out to obtain the influence law of temperature on the uniaxial compressive strength, and the uniaxial creep tests were carried out to obtain the influence law of temperature and stress grade on creep, at − 5, − 10 and − 15 °C. The experimental results show that the creep characteristics of frozen soft soil specimens have obvious fuzzy randomness. The traditional ant colony algorithm is improved by optimizing the pheromone fuzzification coefficient, which improves the search efficiency and avoids the local optimum effectively. Subsequently, the improved fuzzy ant colony algorithm is used to invert the flexibility parameters of commonly used permafrost creep models. The fuzzy weight of evaluation index and the fuzzy random evaluation matrix were determined to evaluate the optimal creep model under three different stress levels of frozen soft soil. Finally, the reliability of the fuzzy random evaluation method was verified by engineering measured data.

## Introduction

China's urbanisation rate has continuously increased in recent years. The migration of population to cities has caused a rapid increase in the urban population, resulting in higher traffic pressure. Therefore, developing urban rail transit has been an effective means of improving urban travel. In the last 20 years, China’s urban rail transit has become one of the longest worldwide. Constructing rail transit has become the top development priority of national transportation, particularly in the coastal open cities with rapid economic development. However, the soil materials in coastal areas are soft and have time-varying characteristics owing to the influence of coastal marine geological conditions^1,2^. In subway excavation, the soil around the tunnel is typically reinforced using the artificial freezing method during construction to isolate groundwater effectively and serve as a temporary support^3^.

Soil frozen by artificial freezing is a highly complex porous building material comprising unfrozen water, ice, mineral particles, and cemented ice, among others. These anisotropic components interact with each other. Influenced by uneven temperature fields and moisture migration, the creep of frozen soil in underground engineering shows apparent randomness and fuzziness. Therefore, it is necessary to understand the creep characteristics of artificially frozen soil, a unique building material, for the safety of the subway tunnel construction by freezing method^4,5^. Moreover, according to the geological characteristics of soft coastal soil, scientifically differentiating and evaluating various creep models to represent the creep process is significant for the stability analysis of frozen tunnel walls. In addition, it is a topic in frozen soil mechanics that has gained substantial research attention^6,7^.

Researchers worldwide have conducted studies on the creep model of frozen soil. Through field investigation and microstructure analysis, Cong et al.^8^ preliminarily discussed the creep failure mechanism of expansive soil slope after freeze–thaw (F–T) cycle and established the creep model of expansive soil used to predict the creep amount of each stage. He et al.^9^ performed a long-term graded loading creep test on salt rock samples. The improved isochronal stress–strain method and steady-state creep rate method were used to determine the long-term strength of salt rock, accurately describing the creep behaviour of salt rock. Zhou et al.^10^ performed scanning electron microscope and graded loading creep tests on the deep soft rock with different magnifications and established a three-element non-linear creep model. The tests showed that the creep model was consistent with the creep test data. Zhu et al.^11^ performed the unloading creep test, analysed the strain development with time under different confining pressures, and established a stress-related Merchant model to describe the unloading creep of soft clay. Guo et al.^12^ modified the Singh–Mitchell creep model by logarithmic function based on the compression test of two kinds of coal gangue. The analysis shows that this model can describe the creep characteristics of coal gangue. Liu et al.^13^ used fractional differential elements rather than the viscous element in the traditional Xiyuan model to obtain the non-linear creep parameters and model of rock. Experiments show that the new model can comprehensively describe the non-linear accelerated creep characteristics of rock. Yao et al.^14^ inverted the creep model parameters through compression and triaxial shear tests to describe the creep process from the primary to the third stage.

Summarising the above research results, researchers typically use the least square, Bayesian analysis, maximum likelihood estimation, and other methods based on random theory for parameter inversion of the creep model^15,16^. Although such methods are simple and easy to use, their inversion efficiency is not high in practical engineering. In terms of the creep model evaluation and selection, the single evaluation index was typically used, or the weight of the evaluation index was given by experience. However, such an evaluation system lacks engineering rationality, and the real optimal model is often unavailable. In addition, most of the current analyses of the artificially frozen soil creep model only consider the randomness of parameters and constitutive relations. They do not consider the ambiguity of this unique building material in deep underground engineering.

Therefore, this study performed a uniaxial test analysis of soft frozen soil layer in subway tunnel engineering in the coastal area. The improved fuzzy ant colony algorithm was used to perform fuzzy random inversion of commonly used frozen soil creep model parameters. Accordingly, a double-index fuzzy random evaluation objective function was established. Combined with the actual working conditions of soft soil layers in subway tunnels in coastal areas, traditional creep models were evaluated comprehensively. Moreover, the optimal models under different conditions were obtained. This analysis was integrated into the intelligent calculation, considering randomness and fuzziness. This study provides a new and more effective method for the uncertainty analysis of artificially frozen soil mechanics.

## Uniaxial test and analysis of soft soil samples

### Specimens and test devices

Metro Line 1 in Nantong, one of the 14 coastal development cities in China, has a total length of 52.37 km, with 27 stations. The tunnel between stations along the underground line is constructed using the freezing method. To ensure that the uniaxial test results are representative of the project, the undisturbed soils used in the test were collected from three typical soft soil layers of the subway tunnel in the project constructed using the freezing method.

**Figure 1:**
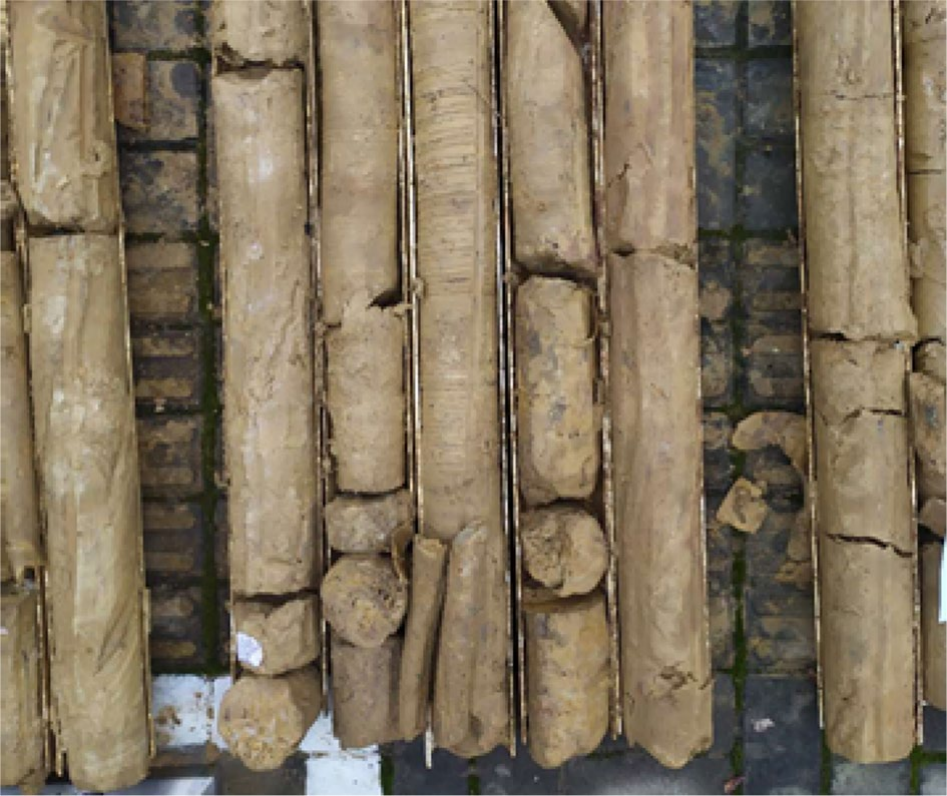
Soil core samples.

In the engineering investigation stage, the hole was turned vertically, the soil core sample was obtained from the corresponding sampling layer (as shown in Fig. 1), and the mud skin was scraped off and carefully sealed with the double-layer plastic preservation package. The sample label was attached to the record, sealed with tape, and tied with a string. The bundled soil sample was placed into the core box, matted with straw and shredded paper, and safely transported to the laboratory^17–19^. Table 1 shows the physical and mechanical parameters of each soil sample layer.

**Table 1:** Primary physical parameters of soil sample layers.

Layer	Soil sample	Depth/m	Water content/%	Dry density/g/cm^3^	Plastic index
1	clay	16.5	21.53	1.50	12.6
2	silt	24.7	18.04	1.57	10.2
3	silt clay	32.3	24.71	1.43	11.8

The geotechnical chamber in the laboratory was opened carefully. The upper and lower layers were distinguished according to the natural deposition direction of soil samples. Subsequently, both ends were sawed flat. According to China's artificially frozen soil test standard (MT/T593.6–2011), the sawed soil samples were made into Φ 50 mm × 100 mm specimens. The shape and parallelism errors were within 1.0% and 0.5 mm, respectively.

**Figure 2:**
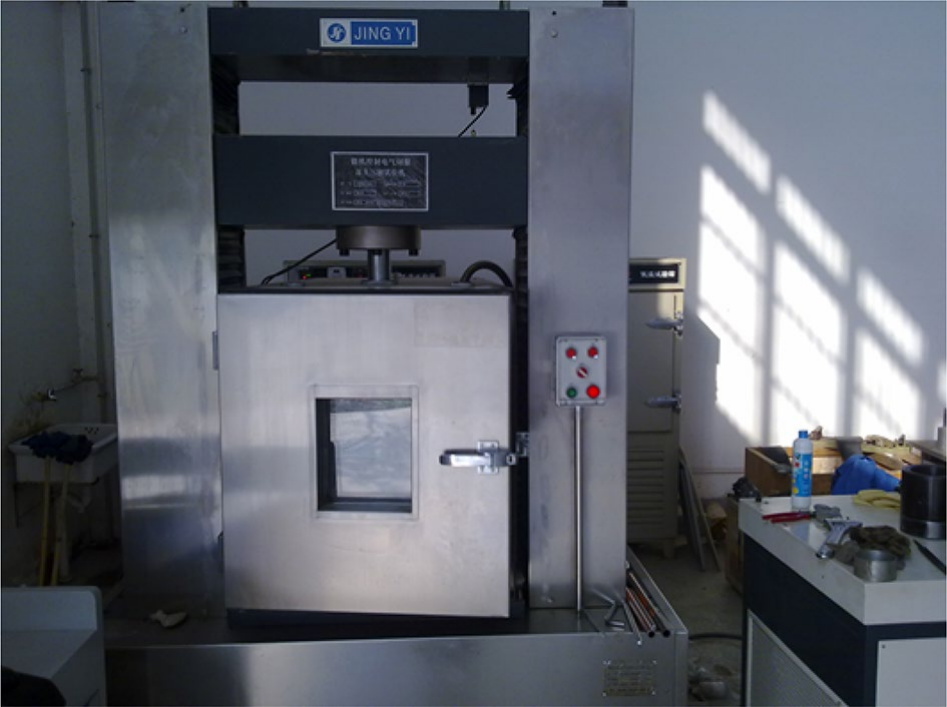
WTD-100 artificially frozen clay apparatus.

The WDT-100 artificially frozen soil test equipment shown in Fig. 2 was used for the uniaxial test. The stress–strain curve can be displayed in real-time in this test. The maximum loading capacity, minimum temperature, and accuracy of the device were 100 kN, − 50 °C, and 1%, respectively. A computer automatically controlled the loading and collected the data according to the set parameters.

### Uniaxial compressive test of frozen soft soil

Before the test, the soft soil specimen was cured at the specified negative temperature for more than 48 h to ensure that the temperature of the specimen was uniform during the test. According to the MT/T593-2011 specification, uniaxial unconfined compressive strength tests of soft soil samples were performed at − 5, − 10, and − 15 °C by strain-controlled loading. Two displacement meters were symmetrically arranged on both sides of the specimen to measure the axial deformation of the specimen and calculate the axial strain by taking the average value^20,21^. During the test, three specimens were used under each temperature condition. Tables 2, 3, 4 show the test results.

**Table 2:** Results of frozen clay uniaxial compressive strength (Layer 1).

Temperature/°C	− 5	− 10	− 15
Uniaxial compressive strength/ MPa	1.73	2.42	3.24
	1.64	2.62	3.13
	1.79	2.50	3.41
Mean/MPa	1.72	2.51	3.26

**Table 3:** Results of frozen silt uniaxial compressive strength (Layer 2).

Temperature/°C	− 5	− 10	− 15
Uniaxial compressive strength/MPa	1.95	3.21	4.22
	2.13	3.19	4.10
	2.28	3.30	4.34
Mean/MPa	2.12	3.23	4.22

**Table 4:** Results of frozen silty clay uniaxial compressive strength (Layer 3).

Temperature/°C	− 5	− 10	− 15
Uniaxial compressive strength/ MPa	2.82	4.13	4.48
	2.46	3.92	4.69
	2.76	3.95	4.82
Mean/MPa	2.68	4.00	4.66

The test results show that the compressive strength of frozen soft soil has a linear relationship with the temperature change under uniaxial compression. The uniaxial compressive strength increased with a decrease in the specimen temperature.

### Stress–strain relationship

**Figure 3:**
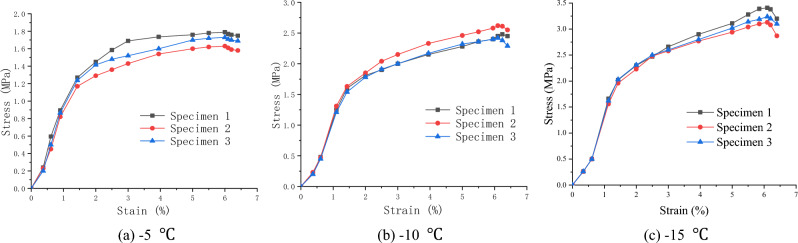
Stress–strain relationship of clay (Layer 1).

**Figure 4:**
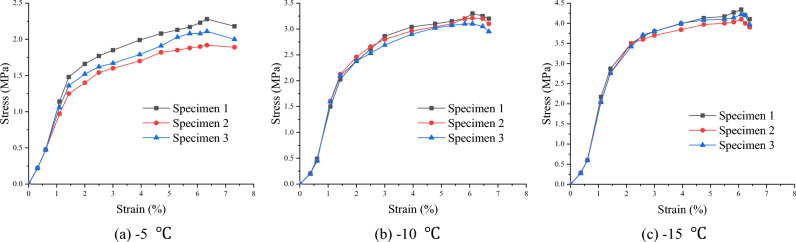
Stress–strain relationship of silt (Layer 2).

**Figure 5:**
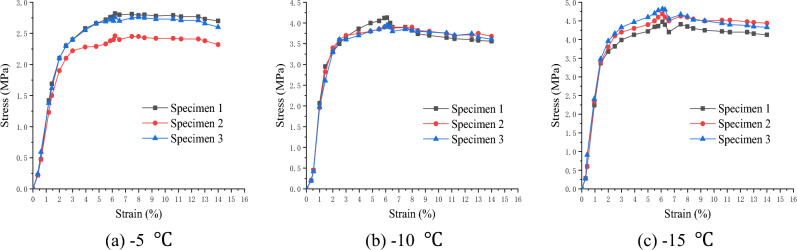
Stress–strain relationship of silt clay (Layer 3).

To describe the stress–strain relationship during the uniaxial compressive test, two displacement meters were arranged symmetrically in the axial direction of the soft soil specimens. Subsequently, the relationship diagrams between axial deformation (strain ε) and load (axial stress σ) of the specimens at different temperatures were established, as shown in Figs. 3, 4, 5.

The test results show that the stress–strain curve of frozen soft soil first exhibited hardening characteristics and then demonstrated a softening trend. The failure deformation was between 10 and 20%, indicating shear expansion failure characteristics.

### Uniaxial creep experiment of frozen soft soil

At the three temperature levels of − 5, − 10, and − 15 °C, the multi-specimen method was used to perform uniaxial creep tests with the stress levels of 0.3 $\sigma_{c}$, 0.5 $\sigma_{c}$ and 0.7 $\sigma_{c}$ respectively, where $\sigma_{c}$ is the uniaxial compressive strength, determined according to Tables 2, 3, 4.

**Figure 6:**
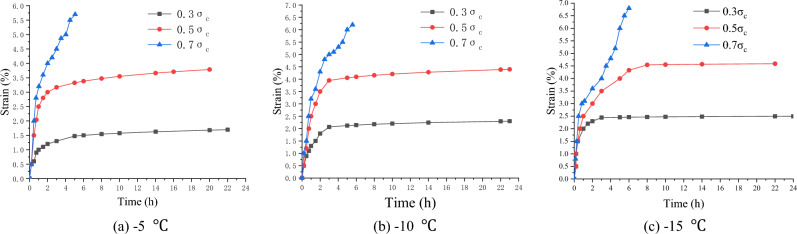
Creep curves of clay (Layer 1).

**Figure 7:**
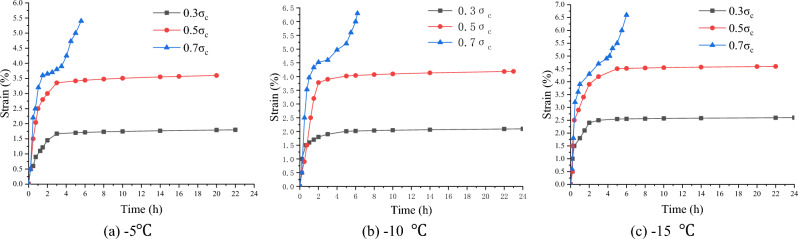
Creep curves of silt (Layer 2).

**Figure 8:**
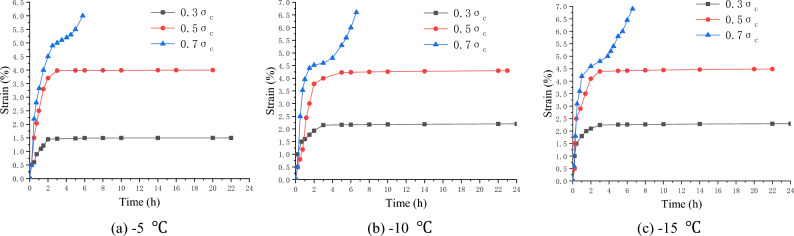
Creep curves of silty clay (Layer 3).

Before the creep test, the specimen was placed between the top and bottom pressure heads of the creep apparatus, and the specimen surface was sealed to prevent changes in the water content. The dynamometer and displacement meter were well installed and connected. Then, the loading system was started, and the specimen was quickly loaded to the required stress level. During the test, the specimen was subjected to a constant stress, and the time and strain values of the whole process were recorded. When the specimens reached stable deformation ($\frac{d\varepsilon }{dt}\le 0.0005{h}^{-1}$) or the deformation rate approached a constant ($\left|\frac{d{\varepsilon }^{2}}{d{t}^{2}}\right|\le 0.0005{h}^{-2}$), the creep tests were stopped^22,23^. Figures 6, 7, 8 show the creep curves.

**Figure 9:**
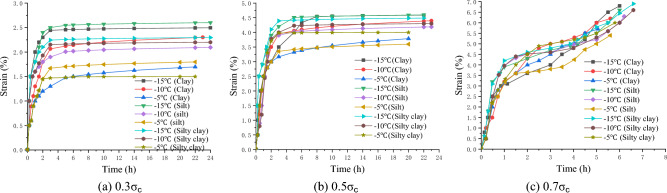
Fuzzy random distribution of creep curves under different stress levels.

The creep value of the frozen specimen decreased with a decrease in the temperature after reaching stability. Under low stress (0.3 $\sigma_{c}$) and medium stress (0.5 $\sigma_{c}$) levels, the entire creep process was in a stable state (stable creep). When the stress level was high (0.7 $\sigma_{c}$), the entire creep process was unstable (accelerated creep). However, from the overall analysis of the test samples, the creep characteristics of the frozen soil samples in the soft soil layer have apparent fuzzy randomness. Figure 9 shows the creep curve fuzzy random distributions under different stress levels.

There are many uncertainties and fuzzy random distributions in actual underground geotechnical engineering. To avoid the limitations of the test and ensure the engineering reliability of the results, this study used a fuzzy random analysis method based on an intelligent calculation to perform effective inversion of frozen soil creep parameters and scientific evaluation of creep models.

## Improved fuzzy random ant colony algorithm

### Traditional ant colony algorithm

In the 1990s, Italian scholar M. Dorigo proposed the ant colony algorithm, an intelligent algorithm developed by simulating the foraging behaviour of real ant colonies in nature, particularly suitable for solving non-linear problems by random search^24–26^.

According to the target constraints, each ant starts from the current city (the city is called the initial state) and follows specific rules to the next city (the city is a feasible solution or part of the solution). In the searching and solving processes, each ant searches for the optimal solution according to the scale characteristics of the problem and the pheromone tracks left by other ants. These trajectories contain heuristic information, telling the ants at the current location the search path of the global solution. According to this scheme, each ant greedily searches for feasible solutions and lists one solution according to the objective constraints as the current optimal solution. However, each ant in the ant colony will have different optimal solutions simultaneously. Accordingly, the global information feedback will be used to make the problem scale evolve toward the global optimal direction and obtain the optimal solution.

However, the traditional ant colony algorithm has some disadvantages when solving actual large-scale problems. For example, the convergence time is long, and the population diversity is difficult to maintain, making the algorithm easy to fall into the local optimal solution, particularly when dealing with fuzzy problems^27^.

### Fuzzy random improvement of ant colony algorithm

The traditional ant colony algorithm was improved to address these limitations^28–30^. The improvements are summarised as follows:

1. At the beginning of the ant colony search, heuristic pheromones are in the accumulation period. During this time, the pheromone gap should not be widened to avoid being trapped in the local optimum. With the initial formation of the pheromone track and increase in iteration times, the gap between pheromones should be increased randomly to avoid the local optimal solution and obtain a better global optimal solution.
2. Previously, pheromones were only updated according to the path travelled by ants in the current optimal solution. The improved fuzzy random ant colony algorithm is based on the current optimal solution of each ant and round counter for fuzzy calculation. Accordingly, the pheromone update amount of each ant is obtained comprehensively.

According to the improvement of the above two aspects, the process of the fuzzy random ant colony algorithm is as follows:

1. Set the number of iterations $Nc$ to 0. The pheromone function $\tau_{ij}$ and increment $\Delta \tau_{ij}^{k}$ are initialised.
2. The starting point set is initialised, and each ant travels from city $i$ to $j$ according to the probability $P_{ij}^{k} (t)$. The city $j$ is then added to the vertex set. Cities to travel next cannot be selected from the elements in the current vertex set, and so on. The travel probability of ants is shown in Eq. (1).
  1 $$P_{ij} (t) = \left\{ {\begin{array}{*{20}c} {\frac{{[\tau_{ij} (t)]^{\alpha } [\eta_{ij} (t)]^{\beta } }}{{\sum\limits_{{s \in J_{k} (i)}} {[\tau_{ij} (t)]^{\alpha } [\eta_{ij} (t)]^{\beta } } }}} & {j \in J_{k} (i)} \\ 0 & {j \notin J_{k} (i)} \\ \end{array} } \right.,$$
  where the random number $\alpha$ is the relative importance of pheromones, $\eta_{{{\text{ij}}}}$ is the heuristic factor, random number $\beta$ is the relative importance of heuristic factors, and $J_{k} (i)$ represents the vertex set that ant k will reach in the next iteration.
3. The objective function of each ant $Y_{k} (k = 1, \cdot \cdot \cdot ,m)$ is calculated according to the specific requirements, and the current optimal solution is recorded at every iteration.
4. The fuzzy calculation is performed according to the current optimal solution of each ant and value of the travel counter, and pheromone updating is considered comprehensively. The updated pheromone amount is shown in Eq. (2).
  2 $$\tau_{ij} (t + n) = \rho \cdot \tau_{ij} (n) + \tilde{c}^{k} \sum\limits_{k} {\Delta \tau_{ij}^{k} } ,$$
  where $\rho (0 < \rho < 1)$ represents the evaporation coefficient of pheromones on the traversal path. $\tilde{c}$ is the optimal pheromone fuzzification coefficient, expressed as follows:
  3 $$\tilde{c} = \frac{{\left| {\tau (Q_{current} ) - \tau (Q_{worst} )} \right|}}{{\left| {\tau (Q_{best} ) - \tau (Q_{worst} )} \right|}},\quad \tilde{c} \in [0,1]$$
  where $\tau (Q_{best} )$, $\tau (Q_{worst} )$, and $\tau (Q_{current} )$ represent the pheromone quantity of the optimal, worst, and current solutions of each travelling ant, respectively.
5. After a round of iteration, the pheromone increment of each side is reset to 0, $Nc \leftarrow Nc + 1$_._
6. If $Nc < Nc_{\max }$ or each ant finds the optimal solution differently, proceed to Step 2 and continue. Otherwise, stop the iteration and find the current optimal solution, which is the global optimal solution.

**Figure 10:**
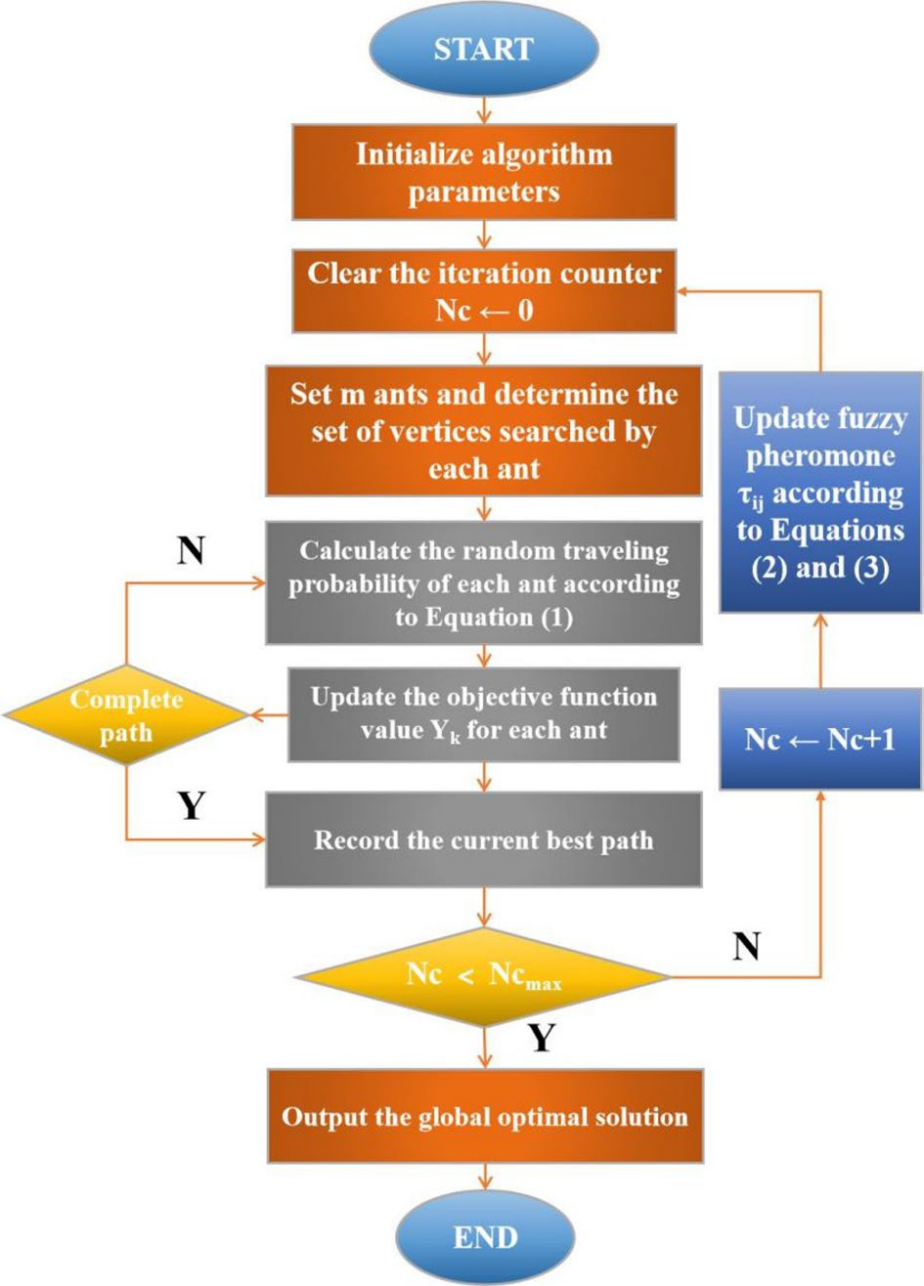
Flow chart of fuzzy random ant colony algorithm.

Figure 10 summarises the flow of the improved fuzzy random ant colony algorithm.

## Fuzzy random analysis of creep model of frozen soft soil

### Creep compliance and compliance parameters

Many previous theoretical and practical studies have shown that the creep of frozen soil is an essential aspect of rheological properties^31^. Unlike plastic deformation, creep does not require the stress to exceed the elastic limit; only if the stress is applied for a long enough time to occur even when the force applied is less than the elastic limit. Therefore, understanding the creep characteristics of frozen soil and effectively determining and studying the creep model is necessary.

**Figure 11:**
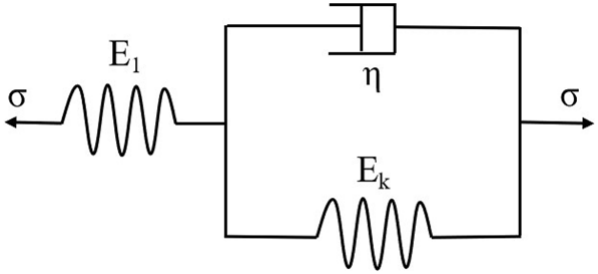
Kelvin model.

Various rock and soil mass creep models can be formed through different series and parallel connections of essential elements, such as springs, sticky pots, and friction plates. For example, the Kelvin creep model is shown in Fig. 11.

According to the principle of superposition, the Kelvin creep equation can be expressed as

4 $$\varepsilon t{ = }\frac{\sigma }{{E_{k} }}\left[ {1 - e^{{ - \frac{{E_{k} }}{\eta }t}} } \right] + \frac{\sigma }{{E_{1} }}$$

where $\sigma$ is the constant stress of the test, t is the action time, E1 is the elastic modulus of spring in the Kelvin model, *E*_k_ is the elastic modulus of the parallel spring in the model, and $\eta$ is the viscosity coefficient of the parallel clay pot. $\eta$ and *E*_1_ are the creep parameters to be retrieved according to different rock and soil conditions. Without loss of generality, all creep equations can be expressed in the following form^32–34^ by considering the primary creep factors and ignoring the minor parameters:

5 $$\varepsilon (t) = \int\limits_{0}^{l} {J(t - \tau )} \frac{d\sigma }{{d\tau }}d\tau$$

Using a differential operator, the creep compliance $J(t)$ is expressed by the following general formula of partial differential equation:

6 $$p_{0} + p_{1} \frac{\partial \sigma }{\partial } + p_{2} \frac{{\partial^{2} \sigma }}{{\partial^{2} }} + \cdots + p_{n} \frac{{\partial^{n} \sigma }}{{\partial^{n} }} = q_{0} \varepsilon + q_{1} \frac{\partial \varepsilon }{\partial } + q_{2} \frac{{\partial^{2} \varepsilon }}{{\partial^{2} }} + \cdots + q_{m} \frac{{\partial^{m} \varepsilon }}{{\varepsilon^{m} }}.$$

The above equation can be simplified as

7 $$P\sigma = Q\varepsilon ,$$

where $$P = \sum\limits_{k = 0}^{n} {p_{k} \frac{{d^{k} }}{{dt^{k} }}}$$, $$Q = \sum\limits_{k = 0}^{m} {q_{k} \frac{{d^{k} }}{{dt^{k} }}}$$_._

The following equation is derived by taking the Laplace transform of the partial differential equation $J(t)$ for creep compliance:

8 $$J(t) = \frac{P(t)}{{tQ(t)}} = \frac{{1 + p_{1} t + p_{2} t^{2} + ... + p_{n} t^{n} }}{{t(q_{0} + q_{1} t + q_{2} t^{2} + ... + q_{m} t^{m} )}}.$$

The Laplace transform of Eq. (9) is continued to derive the final creep compliance expressed as

9 $$J(t) = \varphi (t,\;p,\;q),$$

where $p = \left\{ {p_{1} ,\;p_{2} ,\; \ldots ,\;p_{n} } \right\}$, and $q = \left\{ {q_{0} ,\;q_{1} ,\; \ldots ,\;q_{m} } \right\}$ are the corresponding flexibility parameters.

According to the above methods, the primary creep compliance parameters of several commonly used creep models are shown in Table 5.

**Table 5:** Creep compliance parameters of each creep model.

Creep damage model	Creep compliance parameter
Kelvin	$q_{0} = E_{1} ,q_{1} = \eta$
Generalised Kelvin	$p_{1} = \frac{\eta }{{E_{1} + E_{2} }},q_{0} = \frac{{E_{1} E_{2} }}{{E_{1} + E_{2} }},q_{1} = \frac{{E_{1} }}{{E_{1} + E_{2} }}\eta$
Jeffreys	$p_{1} = \frac{{\eta_{2} }}{{E_{1} }},q_{1} = \eta_{1} + \eta_{2} ,q_{2} = \frac{{\eta_{1} \eta_{2} }}{{E_{1} }}$
Burgers	$p_{1} = \frac{{\eta_{1} }}{{E_{1} }} + \frac{{\eta_{1} + \eta_{2} }}{{E_{2} }},p_{2} = \frac{{E_{1} + E_{2} }}{{E_{1} E_{2} }},q_{1} = \eta_{1} ,q_{2} = \frac{{\eta_{1} \eta_{2} }}{{E_{2} }}$
Nishihara (σ_o_ < σ_s_)	$p_{1} = \frac{\eta }{{E_{1} + E_{2} }},q_{0} = \frac{{E_{1} E_{2} }}{{E_{1} + E_{2} }},q_{1} = \frac{{E_{1} }}{{E_{1} + E_{2} }}\eta$
Nishihara (σ_o_ > σ_s_)	$p_{1} = \frac{{\eta_{2} }}{{E_{1} }} + \frac{{\eta_{1} }}{{E_{2} }} + \frac{{\eta_{2} }}{{E_{2} }},p_{2} = \frac{{\eta_{1} \eta_{2} }}{{E_{1} E_{2} }},q_{1} = \eta_{2} ,q_{2} = \frac{{\eta_{1} \eta_{2} }}{{E_{1} }}$

### Fuzzy ant colony algorithm inversion parameters

According to the uniaxial compression and uniaxial creep test results of frozen soft soil specimens in this study, the deformation trend and data were similar at different temperatures corresponding to the same stress level^35–38^. For example, at − 5 °C, − 10 °C, and − 15 °C, the final strains at different stress levels were as follows: With a 0.3σ_c_ stress level, the final strains of clay were 2.49%, 2.30%, and 1.69%, respectively, those of silt were 2.60%, 2.09%, and 1.79%, respectively, and those of silty clay is 1.50%, 2.29%, and 2.20%, respectively; With a 0.5σ_c_ stress level, the final strains of clay were 4.58%, 4.39%, and 3.79%, respectively, those of silt were 4.59%, 4.18%, and 3.60%, respectively, and those of silty clay were 3.99%, 4.30%, and 4.48%, respectively; and with a 0.7σ_c_ stress level, the final strains of clay were 6.80%, 6.20% and 5.70%, respectively, those of silt were 6.60%, 6.30%, and 5.40%, respectively, and those of silty clay were 6.00%, 6.60%, and 6.90%, respectively. Therefore, taking − 10 °C as an example, a fuzzy ant colony algorithm was used to identify the creep compliance parameters of each model in Table 3 under three stress levels; the rule can be extended to − 5 °C and − 15 °C.

The number of ants was set as $m = 100$, $\alpha = 2$, $\beta = 5$, and $\rho = 0.75$. Subsequently, an ant random parameter given a set of compliance was initialised. The initial information $\tau_{ij}$ and value-added $\Delta \tau_{ij}^{k}$ were calculated using Eq. (2), and the pheromone compliance parameter changes were updated. $\tilde{c}$ is the blur coefficient of the pheromone of the current optimal solution in the travel process. After several iterations of the algorithm, the final global optimal solution was derived as the fuzzy random inversion result of the flexibility parameter, as shown in Table 6.

**Table 6:** Results of fuzzy random inversion of parameters for each creep model.

Creep damage model	Load factor	Parameter inversion result					Coefficient of determination	Number of parameters
		$p_{1}$	$p_{2}$	$q_{0}$	$q_{1}$	$q_{2}$		
Kelvin	0.3	–	–	0.29	0.59	–	0.967	2
	0.5	–	–	0.51	0.33	–	0.934	
	0.7	–	–	0.74	0.28	–	0.881	
Generalised Kelvin	0.3	0.57	–	0.32	0.62	–	0.978	3
	0.5	0.69	–	0.71	0.44	–	0.964	
	0.7	0.81	–	0.93	0.37	–	0.982	
Jeffreys	0.3	0.92	–	–	0.66	0.76	0.894	3
	0.5	1.31	–	–	1.48	0.98	0.970	
	0.7	1.74	–	–	1.69	1.53	0.937	
Burgers	0.3	0.52	1.26	–	0.43	1.13	0.921	4
	0.5	0.84	1.74	–	0.57	1.52	0.972	
	0.7	1.03	2.37	–	1.91	2.86	0.986	
Nishihara	0.3	0.98	–	0.76	0.35	–	0.942	3
	0.5	0.51	–	0.41	0.23	–	0.957	
	0.7	0.72	1.43	–	0.54	1.38	0.995	4
Murayama Shuo Lang	0.3	0.78	0.92	–	0.63	1.47	0.939	4
	0.5	1.19	1.55	–	1.32	1.15	0.948	
	0.7	2.64	1.80	–	2.09	3.83	0.929	

### Improvement of the objective function

Before the improvement, model evaluation in engineering primarily relied on the accuracy index, and the quality of a model was assumed to be completely dependent on its overall calculation accuracy^39,40^. Therefore, the traditional evaluation objective function is expressed as

10 $$\min Y(t) = \sum\limits_{{i = t_{o} }}^{{t_{1} }} {(y_{i} - y_{i}^{\prime } )^{2} } ,\quad t \in (0, + \infty )$$

where $y_{i}$ is the curve fitting value in the case $i$ and $y_{i}^{\prime }$ is the corresponding observed value. The model is optimal when $Y(t)$ obtains the minimum value.

The analysis revealed that evaluating the model from a single index is unreasonable, and assuming a model with high accuracy and complex calculation is unideal^41,42^. Therefore, the model evaluation should adopt a multi-index comprehensive analysis. In this study, the fuzzy random comprehensive evaluation of the creep model was performed based on the dual indexes of measurement coefficient and model algorithm complexity. Subsequently, a new model evaluation objective function was established, changing the previous multi-index evaluation objective function that completely depended on expert experience. Considering that the definition of the evaluation index is ambiguous, the improved fuzzy weighted objective evaluation function of the double index is expressed as follows:

11 $$\min F(n) = \tilde{\omega }_{1} \sum {\mu_{1} R(n)} + \tilde{\omega }_{2} \sum {\mu_{2} O(n)} ,$$

where $\mu_{1} ,\mu_{2}$ are the fuzzy membership functions of each index, $R(n)$ is the measurement coefficient index, $O(n)$ is the complexity index of a model algorithm, and $\tilde{\omega }_{1} ,\tilde{\omega }_{2}$ are the fuzzy weights of each index.

### Fuzzy identification and comprehensive evaluation of creep model

According to the inversion results of the model parameters in Table 6, the generalised Kelvin model was optimal under low stress only considering the measurement coefficient index. The Burgers and westerner models were optimal under medium and high stresses, respectively. The improved objective function of Eq. (11) was used for further comprehensive evaluation. Moreover, the weights of the fuzzy indexes $\tilde{\omega }_{1}$ and $\tilde{\omega }_{2}$ were calculated by combining the two indexes of measured coefficient $R(n)$ and algorithm complexity $O(n)$. The fuzzy comprehensive evaluation matrix was established. Finally, the optimal model under the three stress conditions was comprehensively analysed by fuzzy evaluation.

The measurement coefficient was used to represent the accuracy of the model. The number of parameters was used to represent the complexity of the calculation. The fuzzy evaluation matrix of six commonly used creep models under three stress conditions is expressed as

$$A = \left[ {\begin{array}{*{20}c} 2 & 3 & 3 & 4 & 3 & 4 \\ {0.967} & {0.978} & {{0}{\text{.894 }}} & {{0}{\text{.921 }}} & {{0}{\text{.942}}} & {0.939} \\ \end{array} } \right]$$

$$B = \left[ {\begin{array}{*{20}c} 2 & 3 & 3 & 4 & 3 & 4 \\ {{0}{\text{.934 }}} & {{0}{\text{.964 }}} & {{0}{\text{.970}}} & {{0}{\text{.972 }}} & {{0}{\text{.957}}} & {{0}{\text{.948 }}} \\ \end{array} } \right]$$

$$C = \left[ {\begin{array}{*{20}c} 2 & 3 & 3 & 4 & 4 & 4 \\ {0.881} & {0.982} & {0.937} & {0.986} & {0.995} & {0.929} \\ \end{array} } \right]$$

where A, B, and C are evaluation matrices under low, medium, and high-stress conditions, respectively. The first-row vector of each matrix represents the algorithm complexity of the creep model under the corresponding stress. The second-row vector represents the measured coefficient of the model under the corresponding stress. The matrix column vectors represent the corresponding indexes of the six models.

#### Evaluation matrix dimensionless fuzzification

According to the theory of fuzzy mathematics, it is necessary to normalise the elements of different dimensions of each index in the matrix.

Complexity processing:

12 $$a_{ij} = \frac{{x_{i1} \wedge x_{i2} \wedge \cdots \wedge x_{i6} }}{{x_{ij} }},x_{ij} > 0.$$

Measurement coefficient treatment:

13 $$b_{ij} = \frac{{x_{ij} - (x_{ij} )_{\min } }}{{(x_{ij} )_{\max } - (x_{ij} )_{\min } }},x_{ij} > 0.$$

The normalised fuzzy evaluation matrix is expressed as

$$A^{\prime} = \left[ {\begin{array}{*{20}c} {1.000} & {0.667} & {0.667} & {0.500} & {0.667} & {0.500} \\ {0.869} & {1.000} & {0.000} & {0.321} & {0.571} & {0.536} \\ \end{array} } \right]$$

$$B^{\prime} = \left[ {\begin{array}{*{20}c} {1.000} & {0.667} & {0.667} & {0.500} & {0.667} & {0.500} \\ {0.000} & {0.789} & {0.947} & {1.000} & {0.605} & {0.368} \\ \end{array} } \right]$$

$$C^{\prime} = \left[ {\begin{array}{*{20}c} {1.000} & {0.667} & {0.667} & {0.500} & {0.500} & {0.500} \\ {0.000} & {0.886} & {0.491} & {0.921} & {1.000} & {0.421} \\ \end{array} } \right]$$

#### Determining the fuzzy weight of the evaluation index

First, the mean and standard deviations of each row vector of the three evaluation matrices were calculated using the following equation:

14 $$\overline{x}_{i} = \frac{1}{6}\sum\limits_{j = 1}^{6} {x_{ij} } ,s_{i} = \sqrt {\frac{{\sum\limits_{j = 1}^{6} {(x_{ij} - \overline{x}_{i} )^{2} } }}{5}} ,\quad i = 1,2.$$

Subsequently, the coefficient of variation was calculated using the following equation:

15 $$\tilde{\omega }_{i} = s_{i} /\overline{x}_{i} ,i = 1,2.$$

Finally, the fuzzy weight coefficients under three kinds of stresses were obtained as follows:

Weight of low-stress index: $\tilde{\omega }_{1} = 0.274$, $\tilde{\omega }_{2} = 0.661$.

Weight of medium-stress index: $\tilde{\omega }_{1} = 0.274$, $\tilde{\omega }_{2} = 0.617$.

Weight of high-stress index:$\tilde{\omega }_{1} = 0.305$, $\tilde{\omega }_{2} = 0.623$.

#### Fuzzy random evaluation matrix

The fuzzy random evaluation matrix D row vector can be obtained by multiplying the standardised fuzzy evaluation matrix by the corresponding fuzzy weight of the evaluation index.

$$\begin{aligned} {\text{D1}} & = \left[ {\begin{array}{*{20}c} {\tilde{\omega }_{1} } & {\tilde{\omega }_{2} } \\ \end{array} } \right] \times {\text{A}}^{\prime } = \left[ {\begin{array}{*{20}c} {0.274} & {0.661} \\ \end{array} } \right] \times \left[ {\begin{array}{*{20}c} {1.000} & {0.667} & {0.667} & {0.500} & {0.667} & {0.500} \\ {0.869} & {1.000} & {0.000} & {0.321} & {0.571} & {0.536} \\ \end{array} } \right] \\ & = \left[ {\begin{array}{*{20}c} {0.848} & {0.843} & {0.183} & {0.349} & {0.560} & {0.491} \\ \end{array} } \right] \\ \end{aligned}$$

$$\begin{aligned} {\text{D}}2 & = \left[ {\begin{array}{*{20}c} {\tilde{\omega }_{1} } & {\tilde{\omega }_{2} } \\ \end{array} } \right] \times {\text{B}}^{\prime } = \left[ {\begin{array}{*{20}c} {0.274} & {0.617} \\ \end{array} } \right] \times \left[ {\begin{array}{*{20}c} {1.000} & {0.667} & {0.667} & {0.500} & {0.667} & {0.500} \\ {0.000} & {0.789} & {0.947} & {1.000} & {0.605} & {0.368} \\ \end{array} } \right] \\ & = \left[ {\begin{array}{*{20}c} {0.274} & {0.669} & {0.767} & {0.754} & {0.556} & {0.364} \\ \end{array} } \right] \\ \end{aligned}$$

$$\begin{aligned} {\text{D}}3 & = \left[ {\begin{array}{*{20}c} {\tilde{\omega }_{1} } & {\tilde{\omega }_{2} } \\ \end{array} } \right] \times {\text{C}}^{\prime } = \left[ {\begin{array}{*{20}c} {0.305} & {0.623} \\ \end{array} } \right] \times \left[ {\begin{array}{*{20}c} {1.000} & {0.667} & {0.667} & {0.500} & {0.500} & {0.500} \\ {0.000} & {0.886} & {0.491} & {0.921} & {1.000} & {0.421} \\ \end{array} } \right] \\ & = \left[ {\begin{array}{*{20}c} {0.305} & {0.755} & {0.509} & {0.726} & {0.776} & {0.415} \\ \end{array} } \right] \\ \end{aligned}$$

The final fuzzy random evaluation matrix D was obtained using the improved objective function.

$$ D = \left[ {\begin{array}{*{20}c}    {0.848} & {0.843} & {0.183} & {0.349} & {0.560} & {0.491}  \\    {0.274} & {0.669} & {0.767} & {0.754} & {0.556} & {0.364}  \\    {0.305} & {0.755} & {0.509} & {0.726} & {0.776} & {0.415}  \\   \end{array} } \right] $$

The row vector of fuzzy random evaluation matrix D represents the fuzzy random comprehensive evaluation index of the creep model under low, medium, and high-stress conditions. The column vectors represent six commonly used creep models. According to the maximum fuzzy membership degree principle, the results show that the Kelvin, Jeffreys, and Nishihara models were optimal under low, medium, and high stresses, respectively. The evaluation result is different from that of a single index.

### Efficiency analysis of fuzzy ant colony algorithm

**Figure 12:**
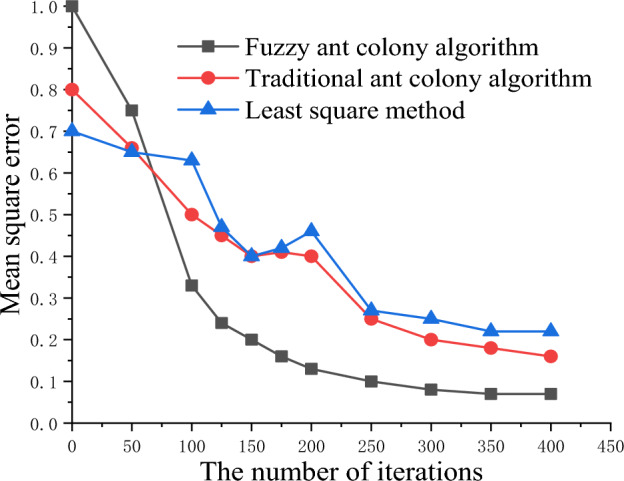
Comparison chart of algorithm efficiency.

Through simulations, the fuzzy ant colony algorithm, traditional ant colony algorithm, and least square method were used to invert the flexibility parameters of the Kelvin model. The inversion efficiencies of the three algorithms were compared. The experimental platform host configuration was as follows: Intel Xeon E-2224G processor, 32G memory, 2TG hard disk, and 1000 M network card, Red Hat Linux 9.0 software platform, and MATLAB 2021A debugging software. Figure 12 shows the test results.

The results show that the fuzzy ant colony algorithm converged faster with an increase in the number of iterations, reducing the error. The fuzzy ant colony algorithm is more robust, convergent, and efficient than other algorithms.

### Comparison between creep model values and engineering test values

**Figure 13:**
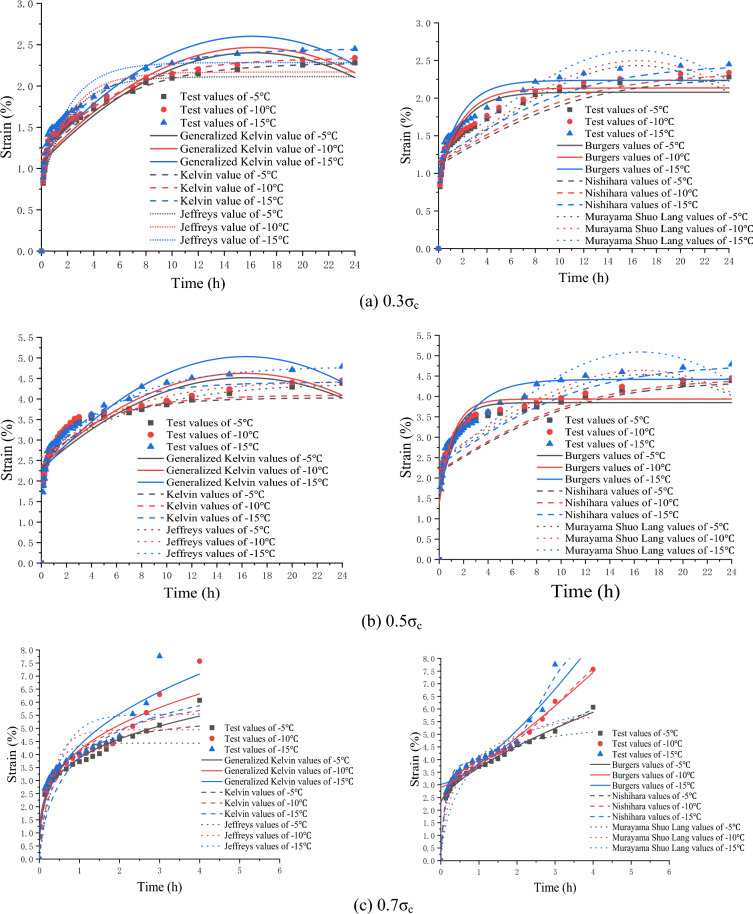
Comparison of various creep constitutive model values with engineering test values.

To verify the conclusion of the fuzzy random evaluation of the creep model, soft soil layers with similar working conditions in the construction project of Nantong Metro Line 2 were selected as verification test materials. The creep test of frozen soil was performed according to the test methods and specifications mentioned above. The creep constitutive model values at different temperatures and stress levels were compared with the engineering test values. Figure 13 shows the results.

The comparison results show that the creep model values after parameter optimization are close to the test values under different temperature and stress conditions. Among them, the Kelvin, Jeffreys and Nishihara model values fit the test values best under low, medium and high stress conditions, respectively. These results are consistent with the conclusion obtained from the fuzzy random comprehensive evaluation in Sect. 3.4.3. This proves that the fuzzy random evaluation method of the creep model of frozen soft soil optimised in this study is reasonable.

## Conclusion

A series of uniaxial tests were performed on artificially frozen soft soil during the construction period of the subway tunnel freezing method. The uniaxial compressive strength and creep law were obtained under different temperatures and stress levels. Based on the fuzzy randomness of underground geotechnical engineering, the improved fuzzy ant colony algorithm was used for parameter inversion and model evaluation. The following conclusions were drawn:

1. Under uniaxial compression conditions, the compressive strength of frozen soft soil had a linear relationship with temperature. The uniaxial compressive strength increased with a decrease in temperature. The failure of frozen soft soil primarily exhibited dilatancy failure characteristics. Under uniaxial creep conditions, the creep value of frozen soft soil decreased with a decrease in temperature when it reached stability. Under low and medium stress, the creep was categorised as a stable creep. Under high stress, the creep was categorised as an accelerated creep.
2. The optimised pheromone fuzzification coefficient was used to improve the traditional ant colony algorithm. The improved fuzzy ant colony algorithm was used to perform fuzzy random inversion of the flexibility parameters of the frozen soft soil creep model. The improved algorithm is more reasonable, robust, and efficient than the traditional parameter inversion algorithm.
3. The fuzzy weighted objective function with dual indexes was established to perform a fuzzy random evaluation on standard creep models. The comprehensive evaluation with dual indexes shows that the Kelvin, Jeffreys, and Nishihara models were optimal under low, medium, and high-stress conditions, respectively.

## Data Availability

The datasets generated and analyzed during the current study are available from the corresponding author upon reasonable request.
